# Practical Guidance for Diagnosing and Treating Iron Deficiency in Patients with Heart Failure: Why, Who and How?

**DOI:** 10.3390/jcm11112976

**Published:** 2022-05-25

**Authors:** Andrew Sindone, Wolfram Doehner, Nicolas Manito, Theresa McDonagh, Alain Cohen-Solal, Thibaud Damy, Julio Núñez, Otmar Pfister, Peter van der Meer, Josep Comin-Colet

**Affiliations:** 1 Heart Failure Unit, Department of Cardiac Rehabilitation, Concord Repatriation General Hospital, Concord, NSW 2139, Australia; 2 Berlin Institute of Health, Center for Regenerative Therapies (BCRT), Charité—Universitätsmedizin Berlin, 10117 Berlin, Germany; wolfram.doehner@charite.de; 3 Center for Stroke Research Berlin (CSB), Charité—Universitätsmedizin Berlin, 10117 Berlin, Germany; 4 Department of Internal Medicine and Cardiology, Campus Virchow-Klinikum, Charité—Universitätsmedizin Berlin, 10117 Berlin, Germany; 5 German Centre for Cardiovascular Research (DZHK), Partner Site Berlin, 10785 Berlin, Germany; 6 Heart Failure and Transplant Unit, Department of Cardiology, University of Barcelona, Bellvitge University Hospital, 08907 Barcelona, Spain; nml@bellvitgehospital.cat; 7 Bellvitge Biomedical Research Institute (IDIBELL), Hospitalet de Llobregat, 08907 Barcelona, Spain; 8 Department of Cardiology, King’s College Hospital, London SE5 9RS, UK; theresa.mcdonagh@kcl.ac.uk; 9 Department of Cardiology, Universite de Paris, UMRS 942, 75010 Paris, France; alain.cohen-solal@aphp.fr; 10 Lariboisière Hospital AP-HP, 75010 Paris, France; 11 Department of Cardiology, AP-HP, DHU A-TVB and GRC ARI, Henri Mondor University-Hospital, 94000 Créteil, France; thibaud.damy@gmail.com; 12 Cardiology Department, Hospital Clínico Universitario, Universidad de Valencia, INCLIVA 46010, 46010 Valencia, Spain; yulnunez@gmail.com; 13 CIBER Cardiovascular, 28029 Madrid, Spain; 14 Department of Cardiology, University Hospital Basel, University of Basel, 4056 Basel, Switzerland; otmar.pfister@usb.ch; 15 Department of Cardiology, University of Groningen, University Medical Center Groningen, 9713 Groningen, The Netherlands; p.van.der.meer@umcg.nl

**Keywords:** chronic heart failure, ferric carboxymaltose, guidelines, iron deficiency

## Abstract

Iron deficiency (ID) is a comorbid condition frequently seen in patients with heart failure (HF). Iron has an important role in the transport of oxygen, and is also essential for skeletal and cardiac muscle, which depend on iron for oxygen storage and cellular energy production. Thus, ID *per se*, even without anaemia, can be harmful. In patients with HF, ID is associated with a poorer quality of life (QoL) and exercise capacity, and a higher risk of hospitalisations and mortality, even in the absence of anaemia. Despite its negative clinical consequences, ID remains under-recognised. However, it is easily diagnosed and managed, and the recently revised 2021 European Society of Cardiology (ESC) guidelines on HF provide specific recommendations for its diagnosis and treatment. Prospective randomised controlled trials in patients with symptomatic HF with reduced ejection fraction (HFrEF) show that correction of ID using intravenous iron (principally ferric carboxymaltose [FCM]) provides improvements in symptoms of HF, exercise capacity and QoL, and a recent trial demonstrated that FCM therapy following hospitalisation due to acute decompensated HF reduced the risk of subsequent HF hospitalisations. This review provides a summary of the epidemiology and pathophysiology of ID in HFrEF, and practical guidance on screening, diagnosing, and treating ID.

## 1. Introduction

Heart failure (HF) impacts in the region of 26 million people across the world and due to the ageing population its prevalence is still increasing [1]. Although there have been advances to prevent and treat HF, it is still associated with substantial rates of mortality and morbidity as well as diminished patient quality of life (QoL) [1,2]. 

HF is defined as a syndrome characterised by cardinal symptoms, for example fatigue, breathlessness and ankle swelling, which may occur alongside signs including peripheral oedema, increased jugular venous pressure and crackles in the lung [3]. HF is caused be an abnormality of the heart, which may be functional and/or structural, resulting in increased pressure in the heart and/or a deficient cardiac output while resting and/or exercising [3].

Iron deficiency is an important and frequent comorbid condition in patients with HF [4,5,6,7,8,9]. In these patients, it independently predicts mortality and morbidity, and is also associated with impaired exercise capacity and reduced QoL [4,5,6,7,8,9]. The recently updated 2021 European Society of Cardiology (ESC) guidelines on HF acknowledge the importance of iron deficiency among patients with HF and also provide specific recommendations for diagnosing and appropriately treating the condition [3]. However, iron deficiency remains under-recognised and under-treated in clinical practice [10,11,12,13,14], likely due in part to a lack of practical guidance for clinicians that can be easily followed.

There are three main goals when treating patients with HF with reduced ejection fraction (HFrEF): (1) lessening mortality; (2) preventing recurrent hospitalisations due to HF worsening; and (3) improving functional capacity, clinical status and QoL [3]. Clinical trial evidence has shown that correcting iron deficiency with supplementary IV iron addresses two of the aforementioned treatment goals (reducing recurrent hospitalisations due to HF, and improving HF symptoms, functional status, and QoL) [15,16,17,18]. Hence, correction of iron deficiency in patients with HFrEF is recommended to improve these clinical outcomes [3].

The majority of patients with HF are managed primarily by general internal medicine physicians who play a crucial role in screening, diagnosing and subsequently treating iron deficiency. This article aims to provide a summary of iron deficiency in HF, along with practical guidance for its diagnosis and appropriate treatment. It aims to address the frequently asked questions of ‘Why’, ‘Who’, and ‘How’ to diagnose and appropriately treat iron deficiency in patients with HF.

## 2. Why Is Diagnosing and Treating Iron Deficiency in Patients with Heart Failure Important?

### 2.1. Role of Iron and the Impact of Iron Deficiency

**Figure 1:**
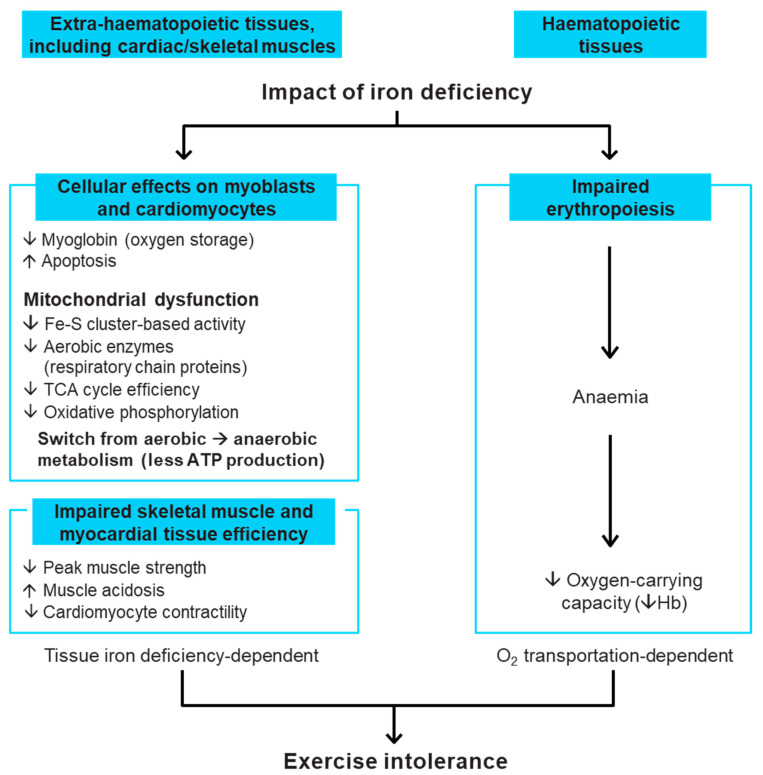
Role of iron in the body and detrimental impact of iron deficiency [20,21,31]. ATP, adenosine triphosphate; Fe-S, iron–sulphur; Hb, haemoglobin; TCA, tricarboxylic acid.

Iron deficiency is a clinical condition where the available iron is inadequate to fulfil the needs of the body [19]. Iron has a critical role in the function of every cell in the human body [7]. As an essential component of respiratory chain proteins in mitochondria, iron is key for cellular energy generation [20]. While iron is most widely recognised for its role in the transport of oxygen as a vital constituent of haemoglobin (Hb), it also has a major role in non-haematopoietic tissues, such as cardiac and skeletal muscle, which are dependent on iron for oxygen storage, mitochondrial energy production and many other cellular processes [20,21] (Figure 1). Thus, iron deficiency *per se*, even in the absence of anaemia (i.e., at a normal Hb level), can be harmful. Experimental studies show that iron deficiency directly weakens the ability of human cardiomyocytes to contract in vitro, and that this can be corrected by iron repletion [22]. In patients who have chronic HF (CHF), iron deficiency can be associated with breathlessness on exertion, increased fatigue, reduced exercise capacity [7,23,24], poorer health-related QoL [25,26], worse HF symptoms, increased HF hospitalisation and higher mortality [5,27,28,29]. These adverse effects are independent of anaemia in patients who have HF and iron deficiency. Furthermore, anaemia does not affect these adverse outcomes in HF when corrected for iron deficiency and other prognostic markers, although patients with both iron deficiency and anaemia have worse outcomes [27,28,29]. Importantly, treatment of iron deficiency with intravenous (IV) iron is associated with improved functional status among patients with HF, even when Hb is normal [15,17,30].

### 2.2. Iron Deficiency Prevalence in Patients with Heart Failure

Iron deficiency is one of the most commonly seen comorbid conditions in patients who have HF, with studies reporting that approximately 40−70% of patients with CHF have iron deficiency [5,7,32,33,34,35,36], regardless of their ejection fraction [9]. Iron deficiency also has a prevalence of up to 80% in patients with acute HF (AHF) [10,37]. Additionally, the prevalence of iron deficiency increases in severe HF (i.e., with higher New York Heart Association [NYHA] class [5]) and when anaemia is present [38].

### 2.3. Iron Deficiency Causes in Patients with Heart Failure

**Figure 2:**
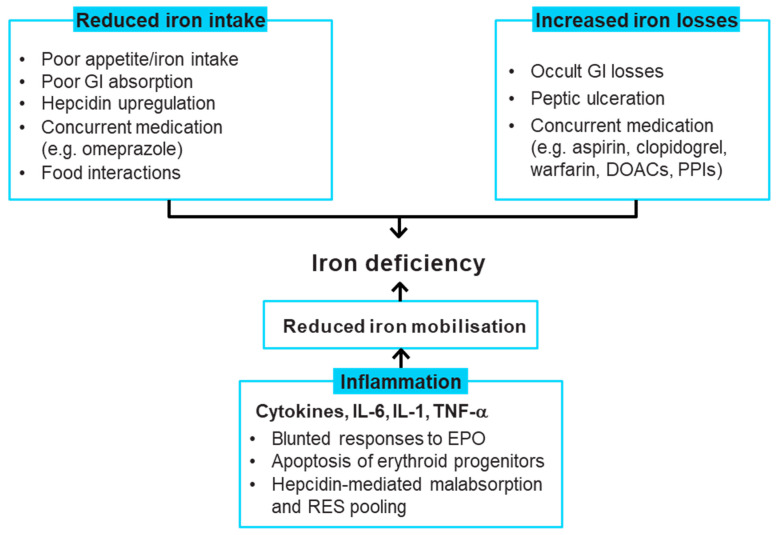
Causes of iron deficiency in heart failure [19,27,29,31,39,40,43,44,45,46]. DOAC, direct oral anticoagulant; EPO, erythropoietin; GI, gastrointestinal; IL, interleukin; PPI, proton-pump inhibitor; RES, reticuloendothelial system; TNF-α, tumour necrosis factor alpha.

The aetiology of iron deficiency in HF is complex and multifactorial, with contradictory evidence on the precise cause(s) [29]. Factors that may contribute to iron deficiency include reduced appetite, co-administration of proton pump inhibitors, occult gastrointestinal blood loss and comorbidities such as chronic kidney disease and inflammatory activity [27,29,39,40]. The possible driving factors for iron deficiency in HF are summarised in Figure 2. Since hepcidin is tightly regulated by inflammatory activation as part of the antibacterial response mechanism and HF is a condition of increased inflammatory activation, patients with HF may have high levels of circulating hepcidin [29,41,42,43]. Hepcidin inhibits iron absorption by binding to ferroportin, causing sequestration of iron in the reticuloendothelial system and reducing the available useable iron [29]. There is some evidence that, as HF progresses and iron deficiency develops, the circulating hepcidin levels may become low in patients with CHF [43,44].

## 3. Who Should Be Assessed for Iron Deficiency?

### 3.1. Who and When to Screen for Iron Deficiency?

**Figure 3:**
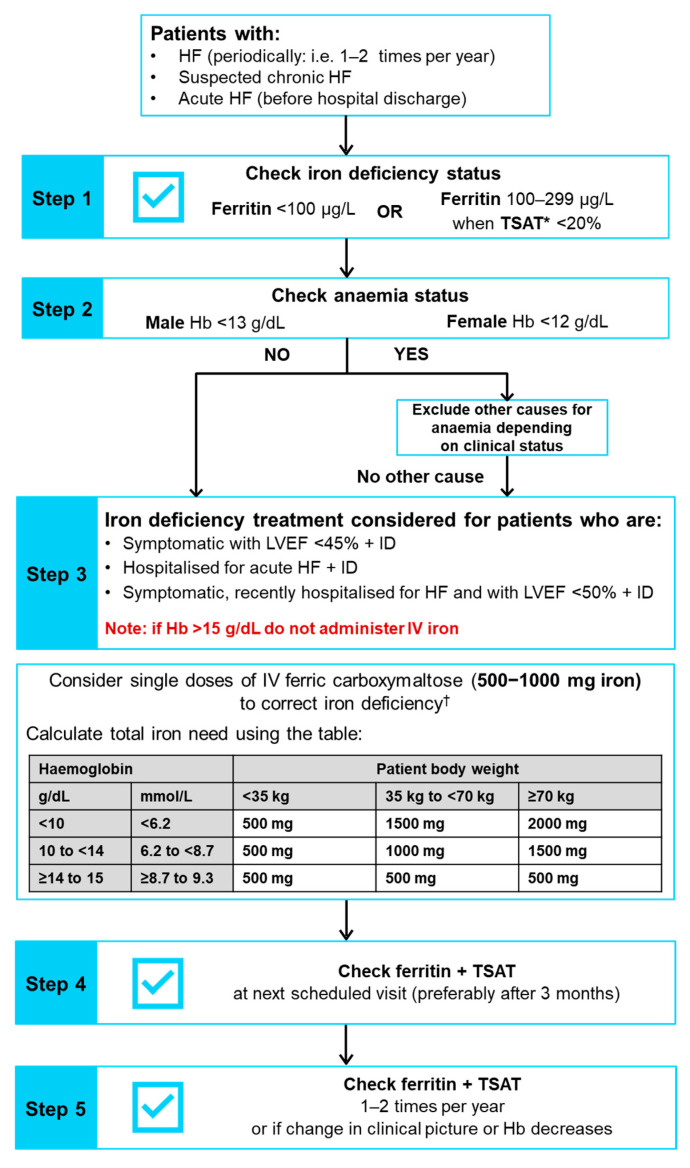
Algorithm showing screening, diagnosing, treating and monitoring for iron deficiency in patients with HF (updated from McDonagh T et al. 2018 [48] in line with the 2021 ESC HF guidelines [3]). * TSAT = (concentration of serum iron/total capacity to bind iron) × 100. ^†^ Note: The use of ferric carboxymaltose has not been assessed in paediatric patients, and therefore treatment with ferric carboxymaltose is not advised in children less than 14 years of age. Full prescribing information can be found in the latest Summary of Product Characteristics [49]. Hb, haemoglobin; HF, heart failure; HFrEF, heart failure with reduced ejection fraction; ID, iron deficiency; IV, intravenous; LVEF, left ventricular ejection fraction; TSAT, transferrin saturation.

The 2021 ESC HF guidelines recommend that every patient with HF should be periodically assessed for iron deficiency (and anaemia) including carrying out a full blood count, and measuring both serum ferritin concentration and transferrin saturation (TSAT) (recommendation class I, evidence level C) [3]; plasma iron level is not an adequate mirror of iron deficiency. This recommendation is a noteworthy update to the 2016 ESC HF guidelines since screening was previously only recommended for new cases of HF [47]. Among the routine blood tests for comorbidities recommended for patients with suspected CHF, iron status (TSAT and ferritin) should also be tested (recommendation class I, evidence level C) [3]. Furthermore, determination of iron status (TSAT and ferritin) is recommended at pre-discharge in patients with AHF [3]. We previously published comprehensive practical recommendations related to diagnosing, treating and monitoring patients with HF and iron deficiency in line with the 2016 ESC HF guidelines [48]. In this article, we have updated our recommendations in line with the 2021 ESC guidelines and recent trial findings, and recommend that clinicians should periodically evaluate iron deficiency and anaemia in all patients with HF regularly as part of the clinical evaluation (i.e., one to two times per year), depending on the iron deficiency severity and HF. Iron status should also be checked in patients with suspected CHF, ambulatory patients or outpatients with worsening HF, and after hospitalisation for AHF. A step-by-step algorithm for screening, diagnosing, treating and monitoring patients with HF is provided in Figure 3.

### 3.2. How to Diagnose Iron Deficiency in Patients with Heart Failure 

Iron status can easily be determined by measuring two readily available blood biomarkers: ferritin and TSAT [19]. Ferritin is a protein for storing iron within cells that is found in every cell type. Serum ferritin concentration is a surrogate marker for the total iron stored in healthy individuals [45]. TSAT is an indicator of the amount of iron circulating in the body that is available to supply metabolising cells and is defined as the percentage (%) of transferrin which is bound to iron [45]. 

In patients with HF, iron deficiency should be diagnosed when serum ferritin is <100 µg/L or TSAT is <20% when serum ferritin is 100–299 µg/L [3]. Two different thresholds are used since serum ferritin may be increased in response to inflammation, such as that seen in CHF, since it is an acute-phase reactant and can therefore appear to fall inside the normal range of 100–300 µg/L [19]. In this situation, a TSAT value of <20% is used to confirm the iron deficiency diagnosis [19]. In line with the 2021 ESC HF guidelines [3], ferritin and TSAT should be assessed at the same time to ensure the correct diagnosis of iron deficiency is made. 

Although lower ferritin thresholds (e.g., <30 µg/L) are used for diagnosis of iron deficiency in other disease areas, it is important to use the thresholds specified above for the diagnosis of iron deficiency in patients who have HF. It is also critical to note that other laboratory parameters, such as mean values of corpuscular volume, corpuscular Hb and corpuscular Hb concentration are not reliable markers of iron deficiency status [50], so should not be used for determining iron deficiency status in patients who have HF. Furthermore, the measurement of only serum iron should not be utilised as an iron deficiency marker, since serum iron concentrations may differ considerably between individual patients with HF and can also display large diurnal fluctuations [51]. When evaluating iron status, it is also important to check for the presence of anaemia, which should be diagnosed using the Hb thresholds of <12 g/dL in females and <13 g/dL in males [52]. 

## 4. How Should Iron Deficiency in Patients with Heart Failure Be Treated? 

Given the serious clinical impact of iron deficiency on patients with HF, it is vital that if diagnosed, this condition is treated. 

### 4.1. Recommendations for Correcting Iron Deficiency

The 2021 ESC HF guidelines recommend that IV FCM should be considered for the treatment of iron deficiency in:

- Symptomatic patients who have a left ventricular ejection fraction (LVEF) < 45% to alleviate symptoms, improve exercise capacity and QoL (recommendation class IIa, evidence level A)
- Pre- and post-discharge follow-up of patients hospitalised for AHF to improve symptoms and reduce rehospitalisation (recommendation class IIa, evidence level B)
- Symptomatic patients recently hospitalised for HF with LVEF < 50% to lessen the risk of HF hospitalisation (recommendation class IIa, evidence level B) [3].

**Figure 4:**
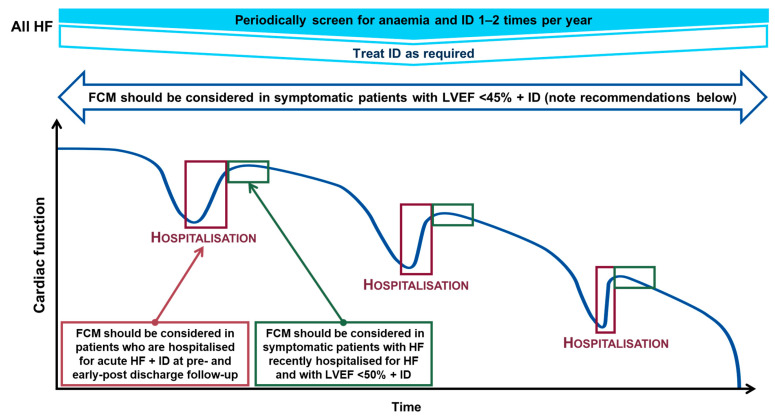
Screening and treatment of iron deficiency across the HFrEF continuum [3,48,53]. Iron deficiency determined by a ferritin <100 μg/L or TSAT <20% when ferritin is 100–299 μg/L; and anaemia determined by a Hb <13 g/dL in males and <12 g/dL in females. TSAT = (serum iron concentration/total iron-binding capacity) × 100. FCM, ferric carboxymaltose; Hb, haemoglobin; HF, heart failure; HFrEF, heart failure with reduced ejection fraction; ID, iron deficiency; LVEF, left ventricular ejection fraction; TSAT, transferrin saturation.

These recommendations were determined from the results of the FAIR-HF, CONFIRM-HF, EFFECT-HF and AFFIRM-AHF trials described in more detail below [15,16,17,18]. A visualisation of the screening and treatment of iron deficiency with FCM across the HFrEF continuum is provided in Figure 4.

### 4.2. Evidence on the Therapeutic Management of Iron Deficiency

Ferric carboxymaltose (FCM), a precision-engineered nanomedicine with a characteristic clinical profile [54], is the most extensively studied IV iron in randomised controlled clinical trials of patients with CHF [15,16,17,18]. Therefore, the majority of the evidence-base for IV iron in HF applies to IV FCM and, as such, FCM is the only iron formulation specifically recommended for the treatment of iron deficiency in the 2021 ESC HF guidelines [3]. 

The largest randomised controlled trials to evaluate FCM in patients who were iron-deficient and had stable CHF (LVEF ≤ 45%) were the FAIR-HF [15], CONFIRM-HF [17], EFFECT-HF [18] and AFFIRM-AHF [16] studies. A summary of the designs and key efficacy and safety findings of these trials is shown in Table 1.

The FAIR-HF study [15] assessed whether treatment with FCM provided a significant improvement of the two primary endpoints: functional capacity as assessed by NYHA functional score and patients’ self-reported perception of wellbeing (Patient Global Assessment [PGA]). This treatment benefit was evident after only 4 weeks of treatment with FCM and was sustained for the duration of the 24-week study. FCM treatment was beneficial for the reduction of HF symptoms, and in improving functional capacity and QoL. The treatment benefits of FCM were comparable among patients either with or without anaemia. FCM was well tolerated, and rates of adverse events, serious adverse events, and death were similar in both the FCM and placebo groups. 

The CONFIRM-HF study [17] evaluated the longer-term efficacy and safety of FCM. In this study, FCM significantly prolonged the Week 24 6-min walk test (6 MWT) distance (a difference of 33 ± 11 metres between the FCM and placebo groups [*p* = 0.002]), and this treatment effect was maintained until Week 52. Patients treated with FCM also achieved benefits to their PGA, NYHA class, QoL and fatigue score, compared with those receiving placebo. These improvements were statistically significant from Week 24 onwards, and the treatment benefits lasted up to 1 year. Patients treated with FCM were also found to have a significantly reduced risk of hospitalisation due to HF worsening compared with those in the placebo group (hazard ratio [HR]: 0.39 [95% confidence interval (CI) 0.19–0.82], *p* = 0.009). The mean dose received by patients was 1500 mg of iron over the 12-month study period, and >75% of the patients needed a total of two injections of FCM for correction and maintenance of iron parameters. Analysis of safety outcomes found that the frequency of adverse events and deaths were comparable between the two treatment groups.

The EFFECT-HF study [18] evaluated whether FCM could improve exercise intolerance, based on the assessment of alteration in peak VO_2_ from baseline to Week 24. FCM had a favourable effect on peak VO_2_, compared with the control (treatment with standard of care), regardless of baseline anaemia status, and also significantly improved PGA score and NYHA functional class of patients in the study. In this study FCM was mostly well tolerated; there were no hypersensitivity reactions to FCM nor cases of hypophosphataemia reported.

Although the initial randomised, placebo-controlled clinical trials established that IV FCM treatment improved symptoms, functional capacity and health-related QoL of HFrEF patients with iron deficiency, they were not planned or sufficiently powered to assess the treatment effects on hard outcomes, such as hospitalisations and mortality. However, meta-analyses of FCM vs. placebo randomised controlled trials of patients with CHF who have iron deficiency, including the CONFIRM-HF and FAIR-HF studies, indicated that IV FCM treatment reduced the risk of all-cause death or cardiovascular (CV) hospitalisation, CV death or HF hospitalisation, and all-cause/CV death or recurrent CV/HF hospitalisations as combined endpoints [30,56]. 

**Figure 5:**
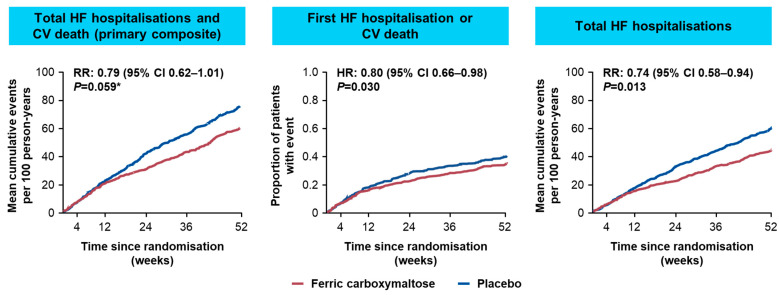
Key primary and secondary outcome results from AFFIRM-AHF [16]. * AFFIRM-AHF primary endpoint narrowly missed statistical significance. AFFIRM-AHF, Study to Compare Ferric Carboxymaltose With Placebo in Patients With Acute Heart Failure and Iron Deficiency; CI, confidence interval; CV, cardiovascular; HF, heart failure; HR, hazard ratio; RR, rate ratio.

Subsequently, the AFFIRM-AHF study evaluated the FCM treatment effect when initiated as early as hospital discharge on mortality and morbidity of patients who were hospitalised due to acute decompensated HF with LVEF < 50% and iron deficiency [16]. Overall, 1108 patients with HF randomised to treatment with FCM (*n* = 558) or placebo (*n* = 550) for up to 24 weeks were considered in the analysis [16]. The study reported 293 primary events in the FCM vs. 372 in the placebo groups (rate ratio [RR]: 0.79, 95% CI 0.62−1.01, *p* = 0.059) for the primary composite endpoint of total hospitalisations for HF and CV deaths for up to 52 weeks, failing to reach the standard statistical significance level of 5% (Figure 5). The secondary endpoint analyses showed that treatment with FCM significantly reduced the risk of HF hospitalisations by 26% compared with placebo (RR: 0.74, 95% CI 0.58–0.94; *p* = 0.013), and this treatment benefit was observed for anaemic and non-anaemic patients. Statistically significant treatment benefits with FCM therapy vs. placebo were also observed for the composite endpoint of time to first HF hospitalisation or CV death (HR: 0.80, 95% CI 0.66–0.98, *p* = 0.03) and for days lost due to HF hospitalisations and CV death (RR: 0.67, 95 CI 0.47–0.97; *p* = 0.035). Additionally, patients in the AFFIRM-AHF study receiving FCM also had significantly greater improvements in the Kansas City Cardiomyopathy Questionnaire (KCCQ) compared with patients receiving placebo: adjusted mean differences (95% CI) at Week 4 were 2.9 (0.5–5.3, *p* = 0.018) for overall summary score (OSS) and 2.8 (0.3–5.3, *p* = 0.029) for clinical summary score (CSS), and at Week 24 were 3.0 (0.3–5.6, *p* = 0.028) for OSS and 2.9 (0.2–5.6, *p* = 0.035) for CSS [57]. Treatment with FCM was well tolerated by patients in AFFIRM-AHF and there were no unexpected safety findings [16].

### 4.3. Safety and Tolerability of FCM

Evidence from clinical trials has shown that FCM is well tolerated by patients with HF and has a favourable safety profile [15,16,17,18]. The most commonly reported adverse drug reactions in patients who received FCM in clinical trials and real-world practice (occurring in ≥1% to 10% patients) were dizziness, flushing, headache, hypertension, hypophosphataemia, injection-/infusion-site reactions and nausea [49]. Anaphylactoid/anaphylactic reactions are rare (≥1/10,000 to <1/1000) and fatalities have been reported [49]. Moderate or severe hypophosphataemia has more commonly been reported in patients treated with FCM within the cardiology therapy area (9.9%) than the neurology and gastroenterology therapy areas (39% and 47.1%, respectively), but hypophosphataemia does not result in serious clinical outcomes for most patients across the populations studied [58]. Although a higher incidence of hypophosphataemia has been reported with FCM in certain patient subgroups [49,58,59,60], such as those who have had a kidney transplantation [60], hypophosphataemia was reported at the same frequency in patients with HF who received FCM or placebo (0.2% in each arm) in the AFFIRM-AHF trial [16]. However, it should be noted that the product label specifies that serum phosphate levels should be monitored in those patients who receive multiple higher-dose injections of FCM or receive FCM long term, and in those patients with pre-existing factors that put them at risk for hypophosphataemia [49]. 

### 4.4. Oral Iron Substitution

Utilisation of oral iron for repletion of deficient iron in patients with HF was specifically evaluated in the 16-week, single, randomised, double-blind, placebo-controlled IRONOUT HF clinical trial [61]. This study assessed the effect of oral iron polysaccharide supplementation at a high dose on exercise capacity among patients with HFrEF (LVEF < 40%) and iron deficiency. Compared with placebo, high-dose oral iron polysaccharide failed to increase exercise capacity, with no significant improvement in the primary endpoint of peak oxygen consumption (peak VO_2_) or in 6 MWT distance over 16 weeks. The study also showed that oral iron polysaccharide therapy provided negligible recovery of stored iron among patients treated with oral iron therapy [61]. Overall, the IRONOUT HF study findings demonstrated that supplementation with oral iron polysaccharide is not an effective strategy for iron deficiency treatment in patients with HFrEF [61], and consequently the 2021 ESC HF guidelines do not recommend oral iron use in patients with HF [3].

### 4.5. Which Patients with Heart Failure Should Receive IV Iron?

FCM treatment benefit has been confirmed by multiple clinical trials in HFrEF [15,16,17,18]. The FAIR-HF, CONFIRM-HF and EFFECT-HF studies involved patients with stable CHF and NYHA class II/III who had a LVEF ≤ 45% [15,17,18]. The AFFIRM-AHF study involved patients with iron deficiency who had an LVEF < 50% and had stabilised following an episode of AHF [16]. A series of prespecified subgroup analyses of the AFFIRM-AHF study showed a consistent effect of FCM on the composite primary outcome across multiple subgroups [16]. While there were interesting observations in terms of the rate ratios when patients were stratified by chronic kidney disease stage, HF aetiology, and HF history, subgroup analyses are of limited power, and therefore, no definitive conclusions can be made on the basis of the subgroup analyses of the AFFIRM-AHF study. 

Little is known about iron deficiency in HF with preserved ejection fraction (HFpEF), and a treatment benefit with IV iron has not been determined in patients with HFpEF since these patients were excluded from previous trials. The aim of the FAIr-HFpEF (NCT03074591) clinical trial, which is currently underway, is to assess the safety and efficacy of IV iron in patients with HFpEF who are iron deficient with or without anaemia (Table 2) [62]. 

It should also be noted that parenteral iron must be used cautiously in cases with acute or chronic infection, asthma, atopic allergies or eczema [49]. Additionally, in patients with ongoing bacteraemia, it is recommended that IV FCM treatment should be stopped. Furthermore, a benefit–risk assessment should be carried out in patients who have a chronic infection which considers erythropoiesis suppression [49].

### 4.6. How to Administer IV Ferric Carboxymaltose and Monitor Patients after Treatment

As previously described [48], administration of IV FCM treatment is based on the patient’s iron need calculated using their weight and Hb (Figure 3 includes a dosing table) [49]. FCM can be administered by IV injection as a slow undiluted bolus (at a rate of 100 mg per minute, or 1000 mg over 15 min), or an infusion that requires dilution [49]. As an infusion, FCM should not be over-diluted to ensure its stability is maintained [49]. The maximum recommended cumulative FCM dose is 1000 mg of iron equivalent to 20 mL FCM per week. IV iron should only be administered in the immediate vicinity of staff trained to assess and treat anaphylactic reactions, and in a location where full resuscitation facilities are available [49,63]. Following every IV iron injection, observation of the patient for any adverse effects is required for a minimum of 30 min [49].

Iron status should then be re-assessed after three months following iron replacement and further repletion provided as required. As indicated, patients should also be evaluated for loss of blood. It is important to avoid early re-assessment of iron status (i.e., occurring within four weeks of the administration of IV iron) since ferritin markedly increases following such administration, and therefore ferritin levels should not be used early on to indicate iron status. In agreement with the 2021 ESC HF guidelines [3], this working group recommends periodically and regularly evaluating iron deficiency and anaemia in all patients with HF as part of clinical evaluation (i.e., one to two times per year, depending on the severity of iron deficiency and HF). Anaemia and iron deficiency should also be evaluated when HF is decompensated, or when symptoms continue even though a patient has received optimised background treatment for HF. IV iron should then be administered as needed. 

### 4.7. Evidence for Erythropoiesis-Stimulating Agent Therapy

The 2021 ESC HF guidelines state that in HF, erythropoiesis-stimulating agent (ESA) treatment of anaemia is not recommended in cases where there are no other indications for this therapy (recommendation class III) [3]. This was determined on the basis of findings from a sizeable randomised clinical trial showing that darbepoetin-alpha did not reduce the risk of HF hospitalisations or all-cause mortality, and the risk of thromboembolic events was found to be increased in patients with HFrEF and mild-to-moderate anaemia [64].

### 4.8. Ongoing Research on IV Irons in HF

Prospective, randomised, controlled clinical trials are currently ongoing to investigate the benefit of IV iron on mortality and morbidity outcomes among patients with HF who have iron deficiency (Table 2), and are expected to read out within the next two years. These include the FAIR-HF2 (NCT03036462) [65], FAIR-HFpEF (NCT03074591) [62], HEART-FID (NCT03037931) [66], IRONMAN (NCT02642562) [67] trials, which are evaluating the effects of IV iron vs. placebo among iron-deficient patients with stable CHF. 

## 5. Conclusions

Iron deficiency is one of the most frequent comorbid conditions in HF and can exist with or without anaemia. Iron deficiency has been recognised as a risk factor for worse outcomes associated with reduced exercise capacity and QoL, worse HF symptoms, and a higher risk for hospitalisations and mortality in patients with HF. Therefore, both the prompt diagnosis and appropriate correction of iron deficiency are crucial. Evidence from prospective randomised clinical trials show that supplementation with IV iron is a highly efficacious treatment in iron-deficient symptomatic patients with CHF. IV iron can provide significant improvements in patients’ functional status, exercise capacity and health-related QoL, as well as lessen the risk of recurrent HF hospitalisations among patients hospitalised due to AHF. The 2021 ESC HF guidelines recommend that IV FCM treatment should be considered in: patients with HF who are symptomatic, have a LVEF < 45% and iron deficiency to improve symptoms of HF, increase exercise capacity and QoL; patients hospitalised for AHF and iron deficiency at pre- and post-discharge follow-up to alleviate symptoms and reduce rehospitalisations; and symptomatic patients who have a LVEF ≤ 50% and iron deficiency recently hospitalised for HF, to reduce the risk of HF hospitalisation [3]. 

## Figures and Tables

**Table 1:** Design and key results from the FAIR-HF, CONFIRM-HF, EFFECT-HF and AFFIRM-AHF clinical trials of IV FCM in patients with HFrEF who have iron deficiency.

	FAIR-HF [15]	CONFIRM-HF [17]	EFFECT-HF [18]	AFFIRM-AHF [16]
Design, duration and number of patients who received treatment per arm	Double-blind, placebo-controlled, randomised; 24 weeks FCM: 305 Placebo: 154	Double-blind, placebo-controlled, randomised; 52 weeks FCM: 152 Placebo: 152	Open-label, SoC-controlled, randomised; 24 weeks FCM: 88 SoC: 86	Double-blind, placebo-controlled, randomised; 52 weeks FCM: 559 Placebo: 551
Key inclusion criteria	NYHA class II (LVEF ≤ 40%) or III (LVEF ≤45%) Hb 9.5–13.5 g/dL ID (ferritin <100 µg/L or 100–299 µg/L + TSAT <20%)	NYHA class II/III (LVEF ≤ 45%) BNP >100 pg/mL and/or NT-proBNP >400 pg/ml Hb <15 g/dL ID (ferritin <100 µg/L or 100–300 µg/L + TSAT < 20%)	NYHA class II/III (LVEF ≤ 45%) BNP >100 pg/mL and/or NT-proBNP >400 pg/ml Hb <15 g/dL ID (ferritin <100 µg/L or 100–300 µg/L + TSAT < 20%) Peak VO_2_ 10–20 mL/kg/min (reproducible)	Hospitalised for acute HF, treated with at least 40 mg IV furosemide (or equivalent) LVEF < 50% ID (ferritin <100 µg/L or 100–299 µg/L + TSAT <20%)
Dosing regimen	Dose determined by Ganzoni formula [55] FCM equivalent to 200 mg iron/week for iron repletion then Q4W for maintenance	FCM equivalent to 500–3500 mg iron for iron repletion (baseline and Week 6); 500 mg iron for maintenance (Weeks 12, 24, 36) if iron deficiency still present	FCM equivalent to 500–1000 mg iron for iron repletion (baseline and Week 6) based on screening Hb and weight; only given at Week 6 if <70 kg and Hb <10 g/dL or ≥70 kg and Hb <14 g/dL; 500 mg iron for maintenance (Week 12) if iron deficiency still present	FCM equivalent to 500–1000 mg at baseline and Week 6 for iron repletion; 500 mg iron for maintenance at Weeks 12 and 24 for patients in whom ID persisted and for whom Hb was 8–15 g/dL
Mean cumulative iron dose/ total number of injections	NA/ Median 6 (3–7) during iron repletion phase	1500 mg/>75% of patients receiving FCM needed 2 injections maximum to correct and sustain iron parameters during the study	1204 mg/42% received 1, 55% received 2, and 3.3% received 3 FCM administrations	1352 mg/80% of patients received 1 or 2 FCM administrations during the treatment phase (i.e., up to Week 24)
Treatment effect on iron-related parameters	FCM vs. placebo at Week 24 (mean ± SE) - Serum ferritin: 312 ± 13 vs. 74 ± 8 µg/L - TSAT: 29 ± 1 vs. 19 ± 1% - Hb: 130 ± 1 vs. 125 ± 1 g/L (*p* < 0.001 for all)	Mean treatment effect (baseline-adjusted) difference for FCM vs. placebo at Week 52: - Serum ferritin: 200 ± 19 µg/L - TSAT: 5.7 ± 1.2% - Hb: 1.0 ± 0.2 g/dL (*p* < 0.001 for all)	FCM vs. control (SoC) at Week 24: - Serum ferritin: 283 ± 150 vs. 79 µg/L - TSAT: 27 ± 8 vs. 20.2% - Hb: 13.9 ± 1.3 vs. 13.2 ± 1.4 g/dL (*p* < 0.05 for all)	Compared with placebo, serum ferritin and TSAT both rose with FCM by week 6 and continued to be significantly higher at week 52
Primary endpoint results	Changes in PGA and NYHA functional class at Week 24 for FCM vs. placebo - PGA: patients reported being much or moderately improved: 50% vs. 28% (OR 2.51; 95% CI, 1.75 to 3.61; *p* < 0.001) - NYHA functional class I/II: 47% vs. 30% placebo (odds ratio for improvement by one class, 2.40; 95% CI, 1.55 to 3.71, *p* < 0.001)	LS means ± SE 6 MWT distance at Week 24 for FCM vs. placebo - 18 ± 8 vs. −16 ± 8 metres (difference FCM vs. placebo: 33 ± 11 metres, *p* = 0.002)	Primary analysis LS means change from baseline in peak VO_2_ at Week 24 for FCM vs. control (SoC) - −0.16 ± 0.387 vs. −1.19 ± 0.389 mL/min/kg (*p* = 0.020) Sensitivity analysis in which missing data were not imputed for control vs. control: - −0.16 ± 0.37 vs. −0.63 ± 0.38 mL/min/kg (*p* = 0.23)	Composite of total HF hospitalisations and CV deaths up to 52 weeks after randomisation for FCM vs. placebo: - 293 primary events (57.2 per 100 patient-years) vs. 372 (72.5 per 100 patient-years) (RR: 0.79, 95% CI 0.62–1.01, *p* = 0.059) - Pre-COVID-19 sensitivity analysis: 274 primary events (55.2 per 100 patient-years) vs. 363 (73.5 per 100 patient-years) (RR: 0.75, 95% CI 0.59–0.96, *p* = 0.024)
Key secondary endpoint results	Significant improvement (*p* < 0.001) with FCM vs. placebo in: - Self-reported PGA at Weeks 4 and 12 - 6 MWT distance at Weeks 4, 12, and 24 - QoL (EQ-5D visual assessment) at Weeks 4, 12, and 24 - Overall KCCQ score at Weeks 4, 12, and 24	Significant improvements in PGA, NYHA class and 6 MWT with FCM vs. placebo: - PGA at Week 12 (*p* = 0.035) Week 24 (*p* = 0.047), Weeks 36 and 52 (both *p* < 0.001) - NYHA class at Week 24 (*p* = 0.004) and Weeks 36 and 52 (both *p* < 0.001) - 6 MWT difference in changes at Week 36 (42 metres with 95% CI of 21–62, *p* < 0.001) and Week 52 (36 metres with 95% CI of 16–57, *p* < 0.001) - Fatigue score at Week 12 (*p* = 0.009), Week 24 (*p* = 0.002) and Week 36 (*p* < 0.001), and Week 52 (*p* = 0.002)	Significant improvements in NYHA class and PGA with FCM vs. control: - NYHA class at weeks 6, 12 and 24 (with imputation; all *p* < 0.05) - PGA at Weeks 12 and 24 (with imputation; *p* < 0.05) Note: effect of FCM vs. control on NYHA class and PGA without imputation (observed values) were similar	Total CV hospitalisations and CV deaths with FCM vs. placebo - 370 vs. 451 (RR: 0·80, 95% CI 0·64–1.00, *p* = 0.050) CV deaths FCM vs. placebo - 77 (14%) vs. 78 (14%) (HR: 0.96, 95% CI 0.70–1.32, *p* = 0.81) Significantly lower number HF hospitalisations with FCM vs. placebo - 217 vs. 294 (RR 0.74; 95% CI 0.58–0.94, *p* = 0.013) Significant treatment benefits with IV FCM vs. placebo for time to first hospitalisation or CV death - 181 (32%) vs. 209 (38%) (HR: 0.80, 95% CI 0.66–0.98, *p* = 0.030)
Safety endpoint results	FCM vs. placebo (incidence per 100 patient-years at risk) - All deaths: 3.4 % vs. 5.5% - Deaths with CV cause: 2.7% vs. 5.5% - Deaths, due to HF worsening: 0% vs. 4.1% - Hospitalisations with CV cause: 10.4% vs. 20.0% - Hospitalisations for worsening HF: 4.1% vs. 9.7%	FCM vs. placebo (incidence per 100 patient-years at risk) - All deaths: 8.9 % vs. 9.9% - Deaths with CV causes: 8.1% vs. 8.5% - Deaths, due to HF worsening: 3.0% vs. 2.1% - Hospitalisations, CV cause: 16.6% vs. 26.3% - Hospitalisations due to worsening HF: 7.6% vs. 19.4%	FCM vs. control (SoC) - All deaths: 0 (0%) vs. 4 (4.7%) - Hospitalisations: 37 (42.0%) vs. 21 (24.4%) ◦ Due to worsening HF: 13 (14.8%) vs. 13 (15.1%) ◦ Due to other CV reason: 13 (14.8%) vs. 3 (3.5%) ◦ Due to non-CV reason: 11 (12.5%) vs. 4 (4.7%)	FCM vs. placebo - Serious adverse events: 250 (45%) vs. 282 (51%) - Cardiac disorder events: 224 (40%) patients with 391 events vs. 244 (44%) patients with 453 cardiac disorder events. - Treatment discontinued prematurely: 157 (28%) vs. 160 (29%) (modified intention-to-treat population)

6 MWT, 6-min walk test; AFFIRM-AHF, Study to Compare Ferric Carboxymaltose With Placebo in Patients With Acute Heart Failure and Iron Deficiency; BNP, brain natriuretic peptide; CONFIRM-HF, Ferric CarboxymaltOse evaluatioN on perFormance in patients with IRon deficiency in coMbination with chronic Heart Failure; CI, confidence interval; CV, cardiovascular; EFFECT-HF, Effect of Ferric Carboxymaltose on Exercise Capacity in Patients With Iron Deficiency and Chronic Heart Failure; EQ-5D, EuroQol-5 Dimension; FAIR-HF, Ferinject assessment in patients with IRon deficiency and chronic Heart Failure; FCM, ferric carboxymaltose; Hb, haemoglobin; HF, heart failure; HFrEF, heart failure with reduced ejection fraction; HR, hazard ratio; ID, iron deficiency; IV, intravenous; KCCQ, Kansas City Cardiomyopathy Questionnaire; LS, least squares; LVEF, left ventricular ejection fraction; NA, not available; NT-proBNP, N-terminal pro B-type natriuretic peptide; NYHA, New York Heart Association; PGA, patient global assessment; Q4W, every four weeks; OR, odds ratio; QoL, quality of life; RR, rate ratio; SE, standard error; SoC, standard of care; TSAT, transferrin saturation.

**Table 2:** Ongoing randomised controlled studies assessing the effect of treatment with IV iron on mortality and morbidity outcomes among patients with HF and iron deficiency.

Study Name	Study Design and Duration	Patient Population/Key Inclusion Criteria	IV Iron Intervention/Dose	Primary Endpoint
FAIR-HF2 (NCT03036462) [65]	Double-blind, parallel-group, randomised, placebo-controlled trial	1200 patients with HFrEF Key inclusion criteria: - Age ≥18 years - CHF for ≥12 months - Iron deficiency	1000 mg FCM followed by optional 500–1000 mg within the first 4 weeks (up to 2000 mg), followed by administration of 500 mg FCM Q4M, unless Hb >16.0 g/dL or ferritin >800 µg/L	Combined rate of HF hospitalisations and CV deaths after ≥12 months of follow-up
FAIR-HFpEF (NCT03074591) [62]	Single-blind, parallel-group, randomised, placebo-controlled trial	200 patients with HFpEF Key inclusion criteria: - Age ≥18 years - LVEF ≥ 45% - Ambulatory ≥7 days with NYHA class II/III - Diuretic treatment - Atrial fibrillation in 2 out of 4 patients - Either hospitalized with an HF diagnosis within 1 year of randomisation or with sinus rhythm and increased plasma natriuretic peptides - Hb >9.0 g/dL and ≤14.0 g/dL - Iron deficiency (ferritin <100 µg/L or TSAT <20% when ferritin 100–299 µg/L)	750 mg FCM given as an infusion over 15 min in 100 mL NaCl	The change in 6-min walking distance measured in meters from baseline to end of study
HEART-FID (NCT03037931) [66]	Double-blind, parallel-group, randomised (1:1), placebo-controlled trial	3068 patients with stable HFrEF Key inclusion criteria: - Age ≥18 years - Stable HF (NYHA class II–IV) on optimal background therapy - LVEF ≤ 40% - Iron deficiency (ferritin <100 µg/L or TSAT <20% when ferritin 100 to 300 µg/L) - Either documented hospitalisation for HF in the past year prior to randomisation OR elevated NT-proBNP within 90 days prior to randomisation	FCM two undiluted bolus doses (15 mg/kg bw) seven days apart to a maximum 750 mg single dose of and a maximum 1500 mg combined dose Q6M as required by iron indices	Composite of: - Incidence of death after 1 year - Incidence of hospitalisation for HF after 1 year - Change in 6 MWT distance at 6 months
IRONMAN (NCT02642562) [67]	Open-label, randomised, standard of care-controlled trial	1300 patients Key inclusion criteria: - Age ≥18 years - LVEF < 45% within the previous 2 years using any conventional imaging modality - NYHA class II–IV - Iron deficiency: ferritin <100 ug/L and/or TSAT < 20% - Evidence of high risk HF with expectation of survival to discharge including hospitalisation for HF currently or within the past 6 months, OR outpatients in atrial fibrillation with NT-proBNP >1000 ng/L or in sinus rhythm with NT-proBNP >250 ng/L (or BNP 300 pg/mL or >75 pg/mL, respectively)	Iron (III) isomaltoside 1000	CV mortality or hospitalisation for worsening HF

6 MWT, 6-min walk test; bw, body weight; CHF, chronic heart failure; CV, cardiovascular; FCM, ferric carboxymaltose; Hb, haemoglobin; HF, heart failure; HFpEF, heart failure with preserved ejection fraction; HFrEF, heart failure with reduced ejection fraction; IV, intravenous; LVEF, left ventricular ejection fraction; NT-proBNP, N-terminal pro b-type natriuretic peptide; NYHA, New York Heart Association; Q4M, every four months; Q6M, every six months; TSAT, transferrin saturation.

## Data Availability

Not applicable.
